# Underestimation Rate in the Percutaneous Diagnosis of Radial Scar/Complex Sclerosing Lesion of the Breast: Systematic Review

**DOI:** 10.1055/s-0041-1741409

**Published:** 2022-01-29

**Authors:** Ana Beatrice Bonganha Zanon, Jonathan Yugo Maesaka, Bruna Bello Chequin, Ana Gabriela de Siqueira Santos, Edmund Chada Baracat, José Roberto Filassi

**Affiliations:** 1 Disciplina de Ginecologia, Departamento de Obstetrícia e Ginecologia, Faculdade de Medicina FMUSP, Universidade de São Paulo, São Paulo, Brazil; 2 Divisão de Ginecologia, Setor de Mastologia, Instituto do Câncer do Estado de São Paulo, Hospital das Clinicas HCFMUSP, Faculdade de Medicina, Universidade de São Paulo, São Paulo, Brazil

**Keywords:** breast diseases, breast neoplasms/diagnosis, image-guided biopsy, doenças mamárias, neoplasias da mama/diagnóstico, biópsia guiada por imagem

## Abstract

**Objective** To evaluate the underestimation rate in breast surgical biopsy after the diagnosis of radial scar/complex sclerosing lesion through percutaneous biopsy.

**Data Sources** A systematic review was performed following the Preferred Reporting Items for Systematic Reviews and Meta-Analyses (PRISMA) recommendations. The Pub **M** ed, SciELO, Cochrane, and Embase databases were consulted, with searches conducted through November 2020, using specific keywords ( *radial scar* **OR** *complex sclerosing lesion* , *breast cancer* , *anatomopathological percutaneous biopsy* **AND/OR** *surgical biopsy* ).

**Data collection** Study selection was conducted by two researchers experienced in preparing systematic reviews. The eight selected articles were fully read, and a comparative analysis was performed.

**Study selection** A total of 584 studies was extracted, 8 of which were selected. One of them included women who had undergone a percutaneous biopsy with a histological diagnosis of radial scar/complex sclerosing lesion and subsequently underwent surgical excision; the results were used to assess the underestimation rate of atypical and malignant lesions.

**Data synthesis** The overall underestimation rate in the 8 studies ranged from 1.3 to 40% and the invasive lesion underestimation rate varied from 0 to 10.5%.

**Conclusion** The histopathological diagnosis of a radial scar/complex sclerosing lesion on the breast is not definitive, and it may underestimate atypical and malignant lesions, which require a different treatment, making surgical excision an important step in diagnostic evaluation.

## Resumo

**Objetivo** Avaliar o grau de discordância entre biópsia percutânea e cirúrgica da mama em pacientes com diagnóstico de cicatriz radiada/lesão esclerosante complexa (CR/LEC) por meio de uma revisão sistemática.

**Fontes dos dados** Foi realizada uma revisão sistemática segundo as recomendações do Preferred Reporting Items for Systematic Reviews and Meta-Analyses (PRISMA, na sigla em inglês). As bases de dados primárias consultadas foram PubMed, SciELO, Cochrane e Embase, com buscas conduzidas até novembro de 2020, utilizando palavras chaves específicas ( *cicatriz radiada* OU *lesão esclerosante complexa* , *câncer de mama* , *anatomopatológico de biópsia percutânea* E/OU *biópsia cirúrgica* ).

**Seleção dos estudos** A busca dos artigos resultou em um total de 584 estudos, sendo 8 selecionados, os quais incluíam mulheres submetidas a biópsia com diagnóstico histológico de CR/LEC e posteriormente submetidas a exérese cirúrgica para avaliar como desfecho o grau de subestimação de lesões atípicas e malignas.

**Coleta de dados** A seleção dos estudos foi conduzida por dois pesquisadores, com experiência na elaboração de revisão sistemática. Os oito artigos selecionados foram lidos na íntegra e submetidos a uma análise comparativa.

**Síntese dos dados** Cicatrizes radiadas/lesões esclerosante complexas foram associadas com lesões atípicas e malignas após a exérese cirúrgica. O grau de subestimação geral foi calculado pela porcentagem de lesões atípicas e malignas no anatomopatológico após a exérese cirúrgica dentre o total de CR/LEC diagnosticadas, enquanto o grau de subestimação de lesões invasoras foi calculado considerando-se apenas os carcinomas invasivos. O grau de subestimação geral dos estudos selecionados variou de 1,3 a 40%, e o de lesões invasoras de 0 a 10,5%.

**Conclusão** O diagnóstico histopatológico de CR/LEC na mama não é definitivo, podendo subestimar lesões atípicas e malignas, cujo tratamento é distinto, tornando a exérese cirúrgica etapa fundamental na investigação diagnóstica.

## Introduction

Radial scar/complex sclerosing lesion (RS/CSL) is a benign breast disease, characterized macroscopically by an architectural distortion of the breast tissue with radial spikes in the center and, microscopically, by a central area of fibroelastosis from which ducts and lobes radiate. 1 2 The distinction between both nomenclatures is based only on the size of the lesion: the radial scar measures < 1 cm and the complex sclerosing lesion is > 1 cm. 2 Most are microscopic, multiple, bilateral, and not palpable on clinical examination.

The implementation of screening programs and the consequent increase in the number of asymptomatic patients undergoing mammography contributed to a 3-fold increase in the detection of these lesions in percutaneous biopsies. 3 Due to its radiological and histological similarity to invasive cancer and its association with other atypical lesions, RS/CSL arouses the interest of researchers; however, the real need for surgical excision is questioned in view of a histopathological diagnosis enabled by percutaneous biopsy.

Once a histological diagnosis is reached after a percutaneous biopsy, the potential for intrinsic malignancy of the lesion must be considered. The lesion can develop into atypical proliferations, including atypical hyperplasia and invasive carcinoma. Besides, its coexistence with cancer and other high-risk lesions should be taken into account. 4 However, the pathogenesis of the lesion, as well as the reason the radial scar is associated with an increased risk of breast cancer, is still uncertain. 3

Hence, the present study aims to assess the degree of disagreement between percutaneous and surgical biopsies in patients diagnosed with RS/CSL through the underestimation rate of atypical and malignant lesions diagnosed after surgical excision.

## Methods

**Fig. 1:**
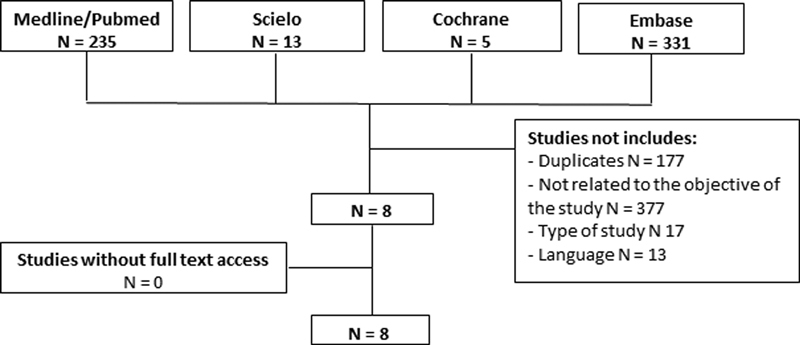
Flow chart following the recommendations of PRISMA.

We conducted a systematic review for the assessment of the underestimation degree of malignant lesions based on a histological diagnosis of RS/CSL lesion after surgical excision and percutaneous biopsy. Studies evaluating RS/CSL with atypia by means of percutaneous biopsy were not included. The review followed the Preferred Reporting Items for Systematic Reviews and Meta-Analysis (PRISMA) guidelines ( Fig. 1 ). 5 The recommendations were adapted from observational studies given the lack of clinical trials.

Pubmed/Medline: *radial scar* [All Fields] OR *complex sclerosing lesions* [All Fields] AND ( *breast neoplasms* [MeSH Terms] OR ( *breast* [All Fields] AND *neoplasms* [All Fields]) OR *breast neoplasms* [All Fields] OR ( *breast* [All Fields] AND *cancer* [All Fields]) OR *breast* *cancer* [All Fields]) AND ( *pathology* [Subheading] OR *pathology* [All Fields] OR *biopsy* [All Fields] OR *biopsy* [MeSH Terms]). Scielo: *radial scar* . Cochrane: *radial scar* . Embase: ( *radial scar* / *exp* OR *radial* *scar* OR *complex sclerosing lesions* ) AND ( *breast* *cancer* / *exp* OR *breast* *cancer* OR ( *breast* / *exp* OR *breast* ) AND ( *cancer* / *exp* OR *cancer* ))) AND ( *biopsy'* / *exp* OR *biopsy* ). Article search and selection were conducted by two researchers experienced in preparing systematic reviews (Zanon A. B. B. and Maesaka J. Y. ), with searches conducted through November 2020. Discrepancies in the selection of articles by these researchers were solved through group discussion with the participation of a third researcher (Chequin B. B.).

## Results

The process of searching, identifying, and selecting articles is in Fig. 1 . From a total of 584 articles, 8 studies were selected for inclusion in the final analysis. The main reasons for exclusion were the following: studies unrelated to the main objective of our review (female patients who underwent biopsy with a resultant histological diagnosis of RS/CSL and subsequently underwent surgical excision, enabling assessment of the underestimation degree of atypical and malignant lesions), type of studies (original studies only), duplicate articles, and articles not written in English or Portuguese. The 8 studies included 630 cases of RS/CSL on percutaneous biopsy, 442 of which subsequently underwent surgical excision ( Table 1 ). The mean age of the patients was 54 years old (range: 19 to 84 years old). The overall underestimation rate was calculated as the percentage of atypical and malignant lesions in the anatomopathological exam of the RS/CSLs that underwent surgical biopsy. The underestimation rate among the studies varied from 1.3 to 40% ( Table 2 ). The invasive lesion underestimation rate was calculated considering only invasive carcinomas in the anatomopathological exam of the RS/CSL that underwent surgical biopsy. The rate varied from 0 to 10.5%.

**Table 1:** Study selection

Author, year of publication	Type of study	RS/CSL diagnosed after percutaneous biopsy	Mean age (years old) (range)	RS/CSL that underwent surgical excision
Quinn et al. (2020) 6	Retrospective	77	54.8 (50–64)	77
Woodward et al. (2020) 7	Retrospective	66	55.6 (19–76)	44
Gašljević et al. (2020) 8	Retrospective	107	61.5 (50–69)	76
Bacci et al. (2019) 9	Retrospective	92	53.4 (25–83)	48
Mooney et al. (2016) 10	Retrospective	54	53.2	25 (46%)
Matrai et al. (2015) 11	Retrospective	77	51.4 (37–79)	77 (100%)
Nassar et al. (2015) 12	Retrospective	100	50.2 (23–74)	38 (38%)
Stefenon et al. (2003) 1	Retrospective	57	49 (31–84)	57(100%)
Final conclusion	−	630	54	442

Abbreviation: RS/CSL, radial scar/complex sclerosing lesion.

**Table 2:** Diagnosis after surgical excision

Author, year of publication	Atypia and LCIS	DCIS	Invasive carcinoma	Overall underestimation rate
Quinn et al. (2020) 6	24/77 (31%)	7/77 (9%)	0/77 (0%)	31/77 (40%)
Woodward et al. (2020) 7	15/44 (34%) 5ADH 1ALH 6FEA 2LCIS 1ADH/ALH	1/44 (2.2%)	1/44 (2.2%)	17/44 (38%)
Gašljević et al. (2020) 8	0/76 (0%)	0/76 (0%)	1/76 (1.3%)	1/76 (1.3%)
Bacci et al. (2019) 9	16/48 (33.3%) 6ADH 6ALH 8FEA	0/48 (0%)	0/48 (0%)	16/40 (33.3%)
Mooney et al. (2016) 10	5/25 (20%) 2ADH 2ALH 1RSA	3/25 (12%)	1/25 (4%) 1ILC	9/25 (36%)
Matrai et al. (2015) 11	9/77 (11.7%) 2ADH 1ALH 6LCIS	0/77 (0%)	0/77 (0%)	9/77 (11.7%)
Nassar et al. (2015) 12	7/38 (18.4%) 1ADH 5ALH 1LCIS	2/38 (5.3%)	2/38 (5.3%)	11/38 (28.9%)
Stefenon et al. (2003) 1	9/57 (15.8%) 5ADH 4ALH	0/57 (0%)	6/57 (10.5%)	15/57 (26.3%)
Final conclusion	85/442 (19.2%)	13/442 (2.9%)	11/442 (2.4%)	109/442 (24.6%)

Abbreviations: ADH, atypical ductal hyperplasia; ALH, atypical lobular hyperplasia; DCIS, ductal carcinoma in situ; ILC, invasive lobular carcinoma; LCIS, lobular carcinoma in situ; FEA, flat epithelial hyperplasia; RSA, radial scar with atypia.

Quinn et al. 6 performed a retrospective review of articles retrieved from a breast screening database at one of the four national units in Ireland. Patients with a radial scar identified on core biopsy or surgical excision were selected. Radial scars without atypia (on core or excision biopsy) were analyzed separately from those with any coexistent risk lesions.

The mean age of the patients was 54.8 years old. There were 425 patients who underwent biopsies; of these, 95 (22%) were found to have RS/CSL on core needle biopsy and 77 had radial scar without evidence of atypia. Analysis of these 77 patients showed that the upgrade to the atypia rate was 31% (24 of 77) and to the carcinoma in situ rate was 9% (7/77). There was no upgrade to invasive carcinoma. Therefore, the overall underestimation rate was 40%. 6

In Woodward et al., 7 a retrospective review using a single institutional pathology and radiology database was conducted for all radial scars identified on core biopsy from January 2010 to January 2017. The mean age of the patients with benign biopsies was 52.9 years old. Sixty-six isolated RSs were identified and 44 underwent surgical excision. Fifteen upgraded to atypical lesions (flat epithelial hyperplasia [6], atypical ductal hyperplasia [5], lobular carcinoma in situ [2], atypical lobular hyperplasia [1], and atypical ductal hyperplasia/atypical lobular hyperplasia [1]) and 2 upgraded to malignancy (ductal carcinoma in situ [1] and invasive ductal carcinoma [1]). The overall underestimation rate was 38% and the invasive lesion underestimation rate was 2.2%. 7

Gašljević et al., 8 in a retrospective study using a database from the Slovenian National Breast Cancer Screening Program, checked all patients with a radial scar or a complex sclerosing lesion who underwent core needle biopsy between 2008 and 2018. The mean age was 61.5 years old. Of the 156 patients selected, 107 (68.6%) had radial scars or complex sclerosing lesions without atypia, and 76 of these patients underwent surgical excision. Seventy-five patients had nonmalignant lesions (atypical proliferative lesions, lobular neoplasia, and papilloma) on final excision and no patient had carcinoma ductal in situ only. One case was upgraded to invasive carcinoma. The overall underestimation rate was 1.3%. 8

Bacci et al. 9 retrieved from an electronic medical record every patient with a histological diagnosis of RS/CSL through vacuum-assisted biopsy at the Comprehensive Cancer Care Centre - Institut Bergonie (France) over a period of 7 years and 5 months, from May 2008 to October 2015. The mean age was 53.4 years old (range: 25 to 83 years old). Ninety-two benign biopsies were identified (biopsies showing isolated RS/CSL or RS/CSL associated with other proliferative lesions without atypia), and 48 lesions underwent surgical excision. After surgery, 16 upgraded to atypia (flat epithelial hyperplasia in 16.7% [8], atypical ductal hyperplasia in 12.5% [6], and atypical lobular hyperplasia in 12.5% [6]). Not a single benign biopsy upgraded to carcinoma in situ or to invasive carcinoma. The overall underestimation rate was 33.3%. 9

Mooney et al., 10 in a retrospective review spanning 14.5 years in Los Angeles (USA), analyzed 5,750 results of core biopsy (the needle size was not specified): 462 were high-risk lesions and 54 were radial scars. Of the latter cases, 25 (46%) underwent surgical excision. Of the remaining 29 cases, 12 did not undergo surgical excision due to loss to follow-up, and 17 had documentation recommending radiological follow-up for 6 months. Surgical excision revealed 5 atypical lesions (atypical ductal hyperplasia [2], atypical lobular hyperplasia [2], and radial scar with atypia [1]), 3 ductal carcinomas in situ, and 1 invasive lobular carcinoma. The general underestimation rate was 36%, while the invasive lesion underestimation rate was 4%. Of the high-risk lesions analyzed, the radial scars were less associated with malignant lesions after excision than atypical ductal hyperplasia (odds ratio [OR] = 0.29; *p* = 0.014; confidence interval [CI] = 0.11–0.77, in this cited study, the range was not mentioned). In addition, in the benign results, the number of cases with Breast Imaging Reporting and Data System (BIRADS) < 4B, nodules < 1 cm, and absence of calcifications was lower than that in the malignant results after surgical excision. 10

In Matrai et al., 11 77 radial scar lesions were found in core biopsies, which subsequently underwent surgical excision were identified in New York (USA). Three patients (3/77) were upgraded to atypical lesions (atypical ductal hyperplasia [2] and atypical lobular hyperplasia [1]) and 6 to lobular carcinoma in situ. There was no upgrade to ductal carcinoma in situ or to invasive carcinoma. The overall underestimation rate was 11.7%. Older age was a predictor of higher risk of upgrade in this setting (62.0 versus 49.9 years old, *p* < 0.001). 11

Nassar et al. 12 performed a retrospective chart review of cases of RS/CSL diagnosed by core biopsy from January 1, 1994 to August 31, 2013 in a single institution in Rochester (USA). One hundred RS/CSL were identified, and the mean age of the patients was 50.2 years old. Of the 100 lesions, 38 underwent surgical excision. The median size of the excision was 1.2 cm (69% > 1 cm). The results were the following: 6 atypical lesions (atypical ductal hyperplasia [1] and atypical lobular hyperplasia [5]), 1 lobular carcinoma in situ, 2 ductal carcinomas in situ, and 2 invasive carcinomas, with an overall underestimation rate of 28.9% and an invasive lesion underestimation rate of 5.3%. 12 Of these 11 lesions, all were diagnosed with radial scar: 3 by mammotomy and 8 by core biopsy.

Stefenon et al. 1 reviewed cases from the archives of the Diagnostic Imaging Center and of the Hospital Santa Rita, Vitória, state of Espírito Santo, Brazil, between October 1993 and December 2001. Of the 926 lesions that underwent percutaneous biopsy, 57 were histopathologically diagnosed with RS/CSL. The mean age of the patients was 49 years old (range: 31 to 84 years old). The lesions were palpable in 10 cases. Mammography showed 48 cases of distortion in architecture, 4 of spiculated nodule, 4 of asymmetric density, and 14 of microcalcifications. The diagnosis after surgical excision was 9 atypical lesions (atypical ductal hyperplasia [5] and atypical lobular hyperplasia [4]), 3 ductal carcinomas in situ together with tubular carcinoma (without specification of adjacent or distant location), 2 tubular carcinomas, and 1 invasive carcinoma. The overall underestimation rate was 26.3% and the invasive lesion rate was 1.7%. 1

## Discussion

The behavior of RS/CSL, a benign lesion of the breast, is not well known, owing to its mimicry and possible progression to atypia and cancer. 12 However, complex sclerosing lesion should not be confused with sclerosing adenosis. The latter refers to another type of benign lesion of glandular proliferation.

Many studies have questioned the need for surgical excision since a histopathological diagnosis is also enabled percutaneously. Brenner et al., 2 in 2002, propounded that percutaneous biopsy should only be complemented by surgical excision when there is associated atypical hyperplasia, a biopsy with < 12 fragments, or when mammographic findings are not compatible with the histological diagnosis of RS/CSL. 2 Chou et al., 13 in 2018, in a follow-up of a mean of 32.3 months, found that in only 1.6% of the patients with a percutaneous diagnosis of RS, the disease had developed into invasive carcinoma. Thus, the RS on percutaneous biopsy could be followed-up with clinical monitoring without the need for an excisional biopsy. 13

Similarly, Nassar et al., 12 in 2015, suggested that RS size at image and the biopsy tissue volume sample may be related to the likelihood of underestimation. Of 11 cases upstaged at excision, 8 (73%) of them were not vacuum-assisted. In this study, “upstaging was noted more often in women with RS lesions larger than 1.0cm and in women with worrisome radiologic features.” In this data, the 29% overall underestimation rate supported the role of excisional biopsy in the follow-up of patients with RS/CSL on percutaneous biopsy. 12

Reaching a correct diagnosis is of extreme clinical importance since the postsurgical treatment described in the literature is different for each lesion. The pure RS/CSL requires no additional procedures. In high-risk histological lesions, an association with endocrine therapy is recommended. For these high-risk lesions, the use of tamoxifen (selective estrogen receptor modulator [SERM]) reduces the risk of invasive carcinoma by 49% ( *p* < 0.00001) and of carcinoma in situ by 50% ( *p* < 0.002). Likewise, the use of aromatase inhibitors demonstrated a 49% decrease in the risk of breast cancer (HR = 0.51; CI = 0.39–0.66; *p* < 0.0001). 14 15 In ductal carcinoma in situ, radiotherapy, after conservative surgery with free margins, reduces the risk of recurrence by up to 59% (HR = 0.41; CI = 0.30–0.57; *p* < 0.0001) and the association with tamoxifen for 5 years also contributes significantly to this reduction (HR = 0.71; CI = 0.58–0.88; *p* = 0.002). 16 17 Finally, invasive carcinomas may require adjuvant treatment (radiotherapy or systemic treatment), depending on histological type, molecular subtype, and staging.

The present study showed that a significant portion of percutaneous biopsies with a diagnosis of RS/CSL turned out to be atypical and malignant lesions upon examination after surgical excision, with a general underestimation rate ranging from 1.3 to 40% in the review studies. In 5 of the 8 studies analyzed, the diagnosis of invasive carcinoma after excision was made, so that the invasive lesion underestimation rate ranged from 1.3 to 10.5% in the studies.

The small number of published articles and their statistical variation constitute a limitation of the present study. The variance may be explained by a different patient profile at each center. The similarity in the mean age of the patients in the 8 studies is stated, but there is no mention of the comorbidities of the patients and of the period between percutaneous and surgical biopsies. There is much variability of percutaneous biopsy types performed in each study: some of the diagnoses were obtained by vacuum-assisted biopsies and others by core biopsies, and two studies did not specify needle sizes, nor the methods used. Probably, a vacuum-assisted biopsy should have a lower underestimation rate than a core biopsy, because it yields larger tissue samples. Despite these limitations and considering that, of the 442 RS/CSL which underwent surgical excision, 109 upgraded to atypical and malignant lesions, which amounts to a 24.6% rate, the present review presents relevant data and reinforces the assumption that a diagnosis by percutaneous biopsy alone can underestimate atypical and malignant lesions.

The relatively high underestimation rate shows the fundamental role of surgical exeresis in the confirmation of a diagnosis, which is necessary to define an appropriate treatment plan for each patient.

## Conclusion

The histopathologic diagnosis of a RS/CSL by percutaneous biopsy is not definitive and may underestimate atypical and malignant lesions, as pointed out in the reviewed studies. In the management of RS/CSL diagnosed by percutaneous biopsy, it is important to consider the volume of tissue evaluated. The high underestimation rate identified in the present study shows the role of surgical excision in the management of the patient.
